# Intracystic magnetic resonance imaging in patients with autosomal dominant polycystic kidney disease: features of severe cyst infection in a case–control study

**DOI:** 10.1186/s12882-016-0381-9

**Published:** 2016-11-09

**Authors:** Tatsuya Suwabe, Yoshifumi Ubara, Toshiharu Ueno, Noriko Hayami, Junichi Hoshino, Aya Imafuku, Masahiro Kawada, Rikako Hiramatsu, Eiko Hasegawa, Naoki Sawa, Satoshi Saitoh, Itsuko Okuda, Kenmei Takaichi

**Affiliations:** 1 Department of Nephrology, Toranomon Hospital Kajigaya, 1-3-1 Kajigaya, Takatsu-ku, Kawasaki-shi, Kanagawa-ken 213-0015 Japan; 2 Okinaka Memorial Institute for Medical Research, Toranomon Hospital, Tokyo, Japan; 3 Department of Hepatology, Toranomon Hospital, Tokyo, Japan; 4 Department of Diagnostic Radiology, International University of Health and Welfare, Mita Hospital, Tokyo, Japan

**Keywords:** Magnetic resonance imaging, MRI, Cyst infection, Infected cyst, Autosomal dominant polycystic kidney disease, ADPKD

## Abstract

**Background:**

The purpose of this study was to investigate the usefulness of intracystic MRI features for detection of severe cyst infection that is usually refractory to antibiotic therapy alone in patients with autosomal dominant polycystic kidney disease.

**Methods:**

Seventy-six patients (88 episodes) with positive cyst cultures treated from January 2006 to December 2013 were enrolled as the cases for this case–control study, while 147 patients who continued to attend our hospital from January 2011 to December 2013 and did not have cyst infection diagnosed during that period were enrolled as the controls. Intracystic MRI findings were investigated.

**Results:**

At least one of four intracystic MRI features (high signal intensity (SI) on diffusion-weighted images (DWI), fluid-fluid level, wall thickening, or gas) was found in all of the cases, but such findings were also detected in some controls. Intracystic gas was specific for cyst infection, but its sensitivity was only 1.1 %. A high intracystic SI on DWI showed a sensitivity of 86.4 %, but its specificity was lower at 33.3 %. Both the specificity and sensitivity of a fluid-fluid level or wall thickening were about 80 %. However, the specificity of these MRI features decreased as total liver and kidney volume (TLKV) increased, falling to 65.8 % in patients with organomegaly (TLKV > 8500 cm^3^). A cyst diameter > 5 cm was useful for detecting severely infected cysts that needed drainage, and specificity was increased by combining the other four MRI findings with a cyst diameter > 5 cm.

**Conclusions:**

MRI with DWI was useful for detecting severe cyst infection in ADPKD. While the specificity of MRI alone was not high enough in patients with organomegaly, combining the four MRI features with abdominal pain, sequential MRI changes, or cyst diameter > 5 cm improved detection of severely infected cysts in these patients.

**Electronic supplementary material:**

The online version of this article (doi:10.1186/s12882-016-0381-9) contains supplementary material, which is available to authorized users.

## Background

**Fig. 1:**
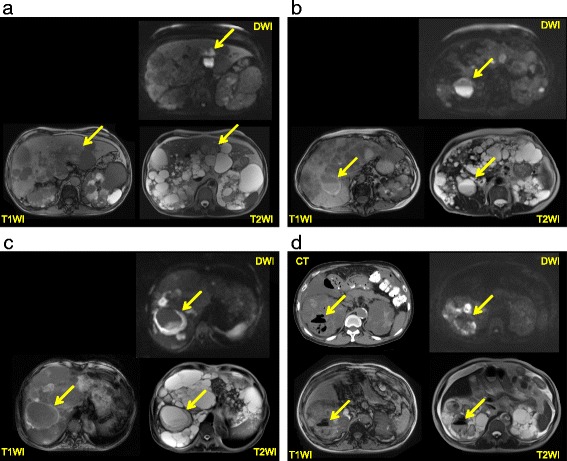
**a** MRI findings (T1WI, T2WI, and DWI) in a patient with cyst infection. The infected renal cyst shows a higher intensity on DWI compared with normal cysts, but it is difficult to identify on T1WI and T2WI. **b** MRI findings (T1WI, T2WI, and DWI) in a patient with cyst infection. A fluid-fluid level and cyst wall thickening can be seen. The infected renal cyst shows a higher intensity on DWI and T1WI than normal cysts, while it has a lower intensity on T2WI. **c** MRI findings (T1WI, T2WI, and DWI) in a patient with cyst infection. Obvious cyst wall thickening can be seen. The infected cyst is iso-intense on T1WI, T2WI, and DWI. **d** MRI findings (T1WI, T2WI, and DWI) in a patient with cyst infection. Gas is seen on T1WI, T2WI, and CT. The infected renal cyst shows a higher intensity on DWI compared with normal cysts, while it has a lower intensity on T2WI and T1WI

Autosomal dominant polycystic kidney disease (ADPKD) is a common inherited renal disorder and the most common monogenetic cause of end-stage renal disease (ESRD) [1]. Cyst infection is a frequent and serious complication of ADPKD that is often difficult to diagnose and treat, and can even be fatal. It has been estimated that 30 % to 50 % of patients with ADPKD experience renal infection during their lifetime [2, 3], although cyst infection leading to hospitalization is much less frequent, occurring in approximately 9 % [4]. Cyst infection often becomes resistant to antibiotics, and percutaneous or surgical drainage of infected cysts is generally recommended when fever persists despite 1–2 weeks of appropriate antimicrobial therapy [1]. It has been reported that infection of large cysts (>5 cm in diameter) is more likely to become severe and that large infected cysts frequently require drainage [4, 5]. However, much is still unknown regarding cyst infection in ADPKD. Positron emission tomography (PET) is useful for detecting infected cysts [4], but this imaging method has the disadvantages of limited availability and high cost. In addition, it was reported that the radiation dose associated with PET might be increased in patients with renal failure [6], making it difficult to perform PET in ADPKD patients whenever cyst infection is suspected. We recently devised MRI diagnostic criteria for cyst infection based on a comparison between cysts with confirmed infection, normal cysts, and cysts with intracystic hemorrhage (Additional file 1) [7]. The possibility of cyst infection can be evaluated by using our criteria, even if causative microorganisms are not identified by culture of the cyst contents [8]. In our diagnostic criteria, detection of four intracystic features on abdominal MRI (a high signal intensity (SI) on diffusion-weighted images (DWI), fluid-fluid level, wall thickening, and gas) and assessment of changes over time are important for detecting cyst infection (Fig. 1a-d), but the sensitivity and specificity of these four features is uncertain. The present study focused on these four intracystic MRI features in ADPKD patients with and without cyst infection to further evaluate the usefulness of our diagnostic criteria.

## Methods

This retrospective case–control study was reviewed and approved by the ethics committee of Toranomon Hospital. All clinical information was obtained from the medical database of Toranomon Hospital (Tokyo and Kawasaki, Japan). We evaluated the sensitivity and specificity of the four intracystic MRI features in our criteria for detecting infected cysts by comparing their frequency between the cases and controls. We also analyzed the MRI features of the cysts in four groups based on total liver and kidney volume (TLKV) quartiles that approximated the quartiles of the controls. We also counted the number of cysts with a diameter >5 cm, because antibiotic therapy is less effective and drainage is more likely to be required for infected cysts with a diameter exceeding 5 cm [4, 5].

### Patients

**Fig. 2:**
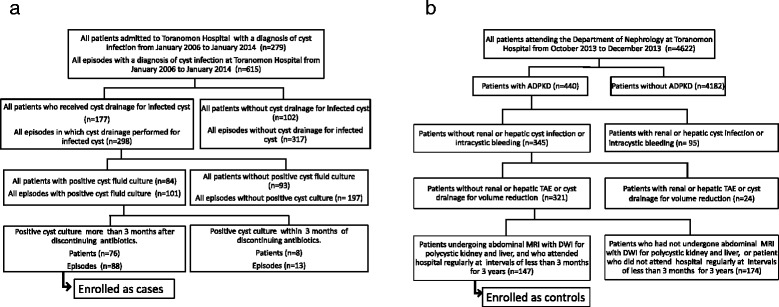
**a** Flowchart of patient inclusion for the cases. **b** Flowchart of patient inclusion for the controls

Only adult patients (20 or more than 20 years old) were enrolled in this study. All patients admitted to Toranomon Hospital with a diagnosis of cyst infection from January 2004 to March 2014 were identified by entering the key words cyst infection for the diagnosis at discharge or the complication during hospitalization. Among them, all patients who underwent cyst drainage and had positive cyst fluid cultures were enrolled as cases in this study. As a result, all of the patients enrolled might have had severe cyst infection since bacteria were not eliminated by prior antibiotic therapy (usually treatment for 1–2 weeks). In order to exclude recurrence of previously undertreated or suppressed cyst infection, we excluded episodes that occurred within 3 months after discontinuing antibiotics for both positive cyst culture (Fig. 2a). All ADPKD patients who continued to attend Toranomon Hospital regularly at intervals of less than 3 months over a period of 3 years (from January 2011 to December 2013) were also investigated for controls. Among them, all patients in whom abdominal MRI with DWI was performed for screening from January 2012 to December 2013 were enrolled as possible controls for this study. Patients with a diagnosis of cyst infection or fever of unknown origin (with or without hospitalization) were excluded from the control group. In addition, patients who received renal or hepatic arterial embolization or cyst drainage for size reduction during the study period were excluded. The controls were selected from among all outpatients attending the Department of Nephrology at Toranomon Hospital from October 2013 to December 2013 according to the procedure shown in Fig. 2b. All of the patients enrolled fulfilled Ravine’s criteria for the diagnosis of ADPKD [9].

### Imaging studies

Abdominal MRI was performed in all patients with suspected cyst infection. In the controls, abdominal MRI was usually performed to evaluate organ volume. We obtained T1-weighted images (T1WI), T2-weighted images (T2WI), and diffusion–weighted images (DWI) in the transverse and sagittal projections in all patients. None of the patients underwent gadolinium-enhanced MRI because of the high prevalence of renal dysfunction. MRI was performed with a 1.5-T apparatus (MagnetomAvanto, Siemens, Erlangen, Germany) and a body phased-array coil. Patients remained in the supine position throughout the examination and all images were obtained with breath holding. T1WI were obtained with the fast low angle shot (FLASH) sequence using the following parameters: repetition time/echo time, 168 ms/2.26 ms; flip angle, 75°; acquisition time, 15 s; number of signals acquired, 1; matrix, 325 × 400 voxels; field of view, 81.25 cm^2^; slice thickness, 9 mm; gap, 1 mm. T2WI were obtained with the half Fourier single shot turbo spin-echo (HASTE) sequence using the following parameters: repetition time/echo time, 1,000 ms/83 ms; flip angle, 75°; acquisition time, 15 s; number of signals acquired, 1; matrix, 256 × 256; field of view, 81.25 cm^2^; slice thickness, 9 mm; gap, 1 mm. DWI were acquired by the single-shot echo-planar imaging sequence with a parallel imaging technique and the following parameters: repetition time/echo time, 2,400 ms/90 ms; acquisition time, 15 s; number of signals acquired, 1; matrix, 350 × 400; field of view, 87.5 cm^2^; slice thickness, 9 mm; gap, 1 mm.

The MRI findings were analyzed by three different readers (a nephrologist, a hepatologist, and a radiologist) who each had more than 10 years of experience. Judgment of the intracystic SI on DWI, the presence of a fluid-fluid level, the cyst wall thickness, intracystic gas, and intracystic changes was done by complete consensus of all three readers. Because the readers had different specialty backgrounds, we did not calculate kappa statistics in this study. The width of each cyst wall was measured and wall thickening was defined as being present when the width was ≥3 mm. Intracystic high SI on DWI was counted even if it was a small cyst whose diameter < 5 mm. Comparison of the SI ratio between infected cysts and skeletal muscle was done as reported previously [7]. If drainage of multiple cysts was performed in a patient, we analysed each cyst separately. We analyzed all of the cysts in each control. If even one of thousands of cysts in a control showed at least four of the specified intracystic MRI features, we judged that as a positive result.

Acute abdominal pain/back pain, gross hematuria, and high intensity on T1WI are suggestive of acute intracystic bleeding. In patients with such symptoms/findings, we usually performed abdominal CT to more clearly differentiate between cyst infection and intracystic hemorrhage. We did not perform drainage of cysts with acute hemorrhage.

### Measurement of kidney and liver volume

Renal volume was calculated on MR images by using the formula for an ellipsoid (*a* × *b* × *c* × π/6), where *a* is the maximum length of the kidney and *b* and *c* are the maximum widths in two transverse dimensions. MR images obtained at a slice interval of 1 cm were analyzed using Synapse software (Fujifilm Company) to measure the liver area (cysts plus parenchyma), and the liver volume was calculated as the sum of the hepatic area on each slice. Then the TLKV was calculated as the sum of the kidney volume and the liver volume.

### Renal function

We classified all of the patients into dialysis and pre-dialysis groups. All of the patients in the dialysis group were on hemodialysis. Patients receive anticoagulation regularly when on hemodialysis, so cyst bleeding could be more likely to occur in this group. The pre-dialysis group was small in cases, so we did not classify it into subgroups according to the glomerular filtration rate.

### Aspiration of infected cysts

Percutaneous aspiration of infected cysts was considered if fever persisted for 1–2 weeks despite appropriate antimicrobial therapy as generally recommended [1]. All infected cysts were identified by abdominal MRI and aspiration was done under ultrasound guidance. A 10.2 Fr pigtail catheter with side holes was inserted percutaneously into each target cyst, after which the contents were aspirated completely and submitted for culture. After cyst drainage, we always performed abdominal CT and injected contrast medium into the cyst through the drain tube to confirm that we had punctured the target cyst. Some patients underwent aspiration of several infected cysts at the same time and the contents of each cyst were cultured.

### Statistical analysis

Results are expressed as the mean ± SD for data analyzed by parametric tests and as the median with interquartile range for data analyzed by non-parametric tests. A probability (P) value of less than 0.05 was considered to indicate significance. For quantitative variables, differences between groups were assessed by Student’s *t*-test, the Mann–Whitney *U* test or the Dunnett test. For quantitative variables, differences between the four groups were assessed by analysis of variance. For categorical variables, differences between groups were assessed by the *χ* ^2^ test or Fisher’s exact test for discrete variables. The usefulness of each diagnostic feature was assessed by receiver operating characteristics (ROC) analysis. Internal validity for area under curve (AUC) of ROC analysis for each diagnostic feature was assessed by calculating the 95 % confidence interval (95 % CI) by the bootstrap resampling technique (2,000 resampling).

All statistical analyses were performed with the SPSS Statistics 18.0 statistical software package (SPSS Inc., Chicago, IL, USA).

## Results

**Fig. 3:**
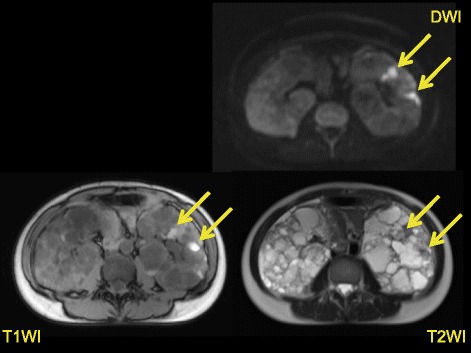
MRI findings (T1WI, T2WI, and DWI) in a control. The renal cysts show a higher intensity on DWI and T1WI than normal cysts, while having a lower intensity on T2WI. These are small cysts with a diameter of less than 5 cm

A total of 279 patients were admitted to our hospital with a diagnosis of cyst infection (615 episodes) during the period from January 2006 to January 2014 (Fig. 2a). Drainage of the infected cyst was performed in 298 episodes and cyst fluid culture was positive in 101 episodes. Among these 101 episodes, 13 episodes with a positive cyst culture obtained within 3 months after discontinuing antibiotics were excluded and there were 88 episodes in total. We sometimes performed drainage of multiple cysts in one patient, so the number of cysts investigated was 110. At least one of the four intracystic MRI features of cyst infection was detected in all 110 cysts by all three readers. However, there was lack of consensus about some findings in a few cysts with multiple intracystic MRI features (three cases of high intracystic SI on DWI and one case of cyst wall thickening). From among the patients who attended Toranomon Hospital regularly, 147 patients were enrolled as controls (Fig. 2b). The cases were significantly older than the controls. TLKV were not significantly different between the cases and the controls (Table 1). A high intracystic SI on DWI was seen in 86.4 % of the cases, but was also found in 66.7 % of the controls (Table 2). In the controls, the majority of cysts with a high intracystic SI on DWI were < 5 cm in diameter (Fig. 3). Intracystic fluid-fluid level and wall thickening were seen in less than 50 % of the cases, but were found in less than 15 % of the controls, so the specificity of these findings was high (>85 %). An intracystic fluid-fluid level or wall thickening were seen in 84.1 % of the cases, and also in 19.7 % of the controls, so the sensitivity and specificity of these findings were about 80 %. Intracystic gas was only seen in 1.1 % of the cases, but its specificity was 100 %. At least one of the four MRI features of cyst infection was detected in all of the cases. In addition, 83.0 % of the cases had cysts with a diameter >5 cm and at least one of these four MRI features of infection, while only 18.4 % of the controls had cysts with a diameter >5 cm and at least one of the four MRI features. In the cases, the frequency of a high intracystic SI on DWI, a fluid-fluid level, and wall thickening was not significantly different among the four TLKV quartiles (Table 3). On the other hand, the frequency of these findings was quite different among the four TLKV quartiles of the controls (Table 4), with the frequency of each finding showing an increase and the specificity becoming lower as the TLKV increased. ROC analysis revealed that a fluid-fluid level or wall thickening were significant in all of the four TLKV quartiles (Table 5). The 95 % CI of each diagnostic feature calculated by the bootstrap resampling technique was similar to that obtained by ROC analysis of raw data, which suggested that internal validity was maintained.

**Table 1:** Clinical characteristics of the subjects

	Cases	Controls	*P* value
Number of episodes (M/F)	88 (39/49)	147 (62/85)	NS
Age [mean±SD]	64.9 ± 10.7	53.3 ± 11.0	< 0.0001
Renal function	Dialysis 71 Pre-dialysis 17	Dialysis 72 Pre-dialysis 75	< 0.0001
Number of cysts studied	110		
Total kidney volume (cm3) [median (IQR)]	1735.6 (1203.5-3319.0)	2246.0 (995.4-4679.1)	<0.05
Total liver volume (cm3) [median (IQR)]	2586.0 (1808.3-4482.7)	1851.1 (1305.5-3409.5)	< 0.05
Total kidney and liver volume (cm3) [median (IQR)]	5275.6 (3224.0-7815.0)	5495.8 (2866.8-8620.7)	NS
Site of infected cyst	Kidney 21, Liver 89		

*SD* standard deviation, *IQR* interquartile range (25 % - 75 %)

**Table 2:** Number of episodes with each MRI feature of cyst infection, and the sensitivity and specificity of each MRI feature

	Cases (*n*=88)	Controls (*n*=147)	Sensitivity	Specificity
High SI on DWI (%)	86.4	66.7	86.4	33.3
Fluid-fluid level (%)	50.0	12.9	50.0	87.1
Wall thickening (%)	48.3	10.9	48.3	89.1
Fluid-fluid level or wall thickening (%)	84.1	19.7	84.1	80.3
Gas (%)	1.1	0	1.1	100
At least one of these four features (%)	100	68.0	100	32.0
High SI on DWI with diameter > 5cm (%)	69.3	15.6	69.3	84.4
Fluid-fluid level or wall thickening with diameter > 5cm (%)	72.7	8.8	72.7	91.2
At least one of these four features with diameter > 5cm (%)	83.0	18.4	83.0	81.6

**Table 3:** Number of episodes in cases with positive MRI features of cyst infection and intracystic changes, and the sensitivity of each MRI feature and intracystic changes in four groups stratified by TKLV

	All episodes in cases (*n*=88)	Cases with TKLV < 3000 cm3 (*n*=13)	Cases with TKLV 3000 to <5500 cm3 (*n*=39)	Cases with TKLV 5500 to 8500 cm3 (*n*=17)	Cases with TKLV >8500 cm3 (*n*=17)	*p*-value
Number of episodes(M/F)	88 (39/49)	13 (0/13)	39 (16/23)	17 (8/9)	17 (14/3)	<0.0001
Age	64.6 ± 10.7	63.4 ± 17.1	65.5 ± 11.0	66.6 ± 7.7	61.8 ± 5.8	NS
Renal function (Dialysis %)	80.2	92.3	76.9	70.6	88.2	NS
High SI on DWI (%)	86.4	92.3	82.5	82.4	94.4	NS
Fluid-fluid level (%)	50.0	38.5	55.0	58.8	38.9	NS
Wall thickening (%)	48.3	53.9	45.0	47.1	52.9	NS
Fluid-fluid level or wall thickening (%)	84.1	84.6	85.0	82.4	83.3	NS
Gas (%)	1.1	0	2.5	0	0	NS
At least one of these four features (%)	100	100	100	100	100	NS
High SI on DWI with diameter > 5cm (%)	69.3	53.9	67.5	82.4	72.2	NS
Fluid-fluid level or wall thickening with diameter > 5cm (%)	72.7	53.9	75.0	82.4	72.2	NS
At least one of these four features with diameter > 5cm (%)	83.0	61.5	85.0	100.0	77.8	NS

*TKLV* total kidney and liver volume, *NS* not significant

**Table 4:** Number of controls without MRI features of cyst infection and intracystic changes, and the specificity of each MRI feature and intracystic changes in four groups stratified by TKLV

	All controls (*n*=147)	Controls with TKLV < 3000 cm3 (*n*=39)	Controls with TKLV 3000 to < 5500 cm3 (*n*=35)	Controls with TKLV 5500 to <8500 cm3 (*n*=35)	Controls with TKLV >8500 cm3 (*n*=38)	*p*-value
Number of patients(M/F)	147 (62/85)	39 (14/25)	35 (14/21)	35 (19/16)	38 (15/23)	NS
Age (years)	53.3 ± 11.0	50.3 ± 14.7	52.3 ± 8.8	55.4 ± 9.5	55.4 ± 9.1	NS
Renal function (Dialysis %)	49.0	5.1	31.4	74.3	86.8	<0.0001
Without high SI on DWI (%)	33.3	69.2	37.1	14.3	10.5	<0.0001
Without fluid-fluid level (%)	87.1	100.0	82.9	82.9	81.6	<0.01
Without wall thickening (%)	89.1	100.0	94.3	82.9	79.0	<0.005
Without fluid-fluid level or wall thickening (%)	80.3	100.0	82.9	71.4	65.8	<0.0001
Without gas (%)	100	100	100	100 %	100	NS
None of the four features (%)	32.0	69.2	34.3	14.3	7.9	<0.0001
Without high SI on DWI (diameter > 5cm) (%)	84.4	100.0	94.3	77.1	65.8	0.0001
Without fluid-fluid level or wall thickening (diameter > 5cm) (%)	91.2	100.0	94.3	94.3	76.3	<0.005
Without at least one of the four features (diameter > 5cm) (%)	81.6	100.0	91.4	74.3	60.5	0.0001

*TKLV* total kidney and liver volume, *NS* not significant

**Table 5:** Results of receiver operating characteristic analysis for each diagnostic feature

		AUC			Bootstrap replicates
		Value	95 % CI	*p*-value	95 % CI of AUC
All episodes	DWI	0.601	0.532 to 0.670	0.006	0.551 to 0.648
	Fluid-fluid level	0.667	0.598 to 0.736	<0.001	0.613 to 0.718
	Wall thickening	0.672	0.604 to 0.741	<0.001	0.621 to 0.724
	Fluid-fluid level or wall thickening	0.805	0.749 to 0.862	<0.001	0.750 to 0.849
	Fluid-fluid level or wall thickening with diameter > 5cm	0.796	0.737 to 0.856	<0.001	0741 to 0.842
Patients with TKLV < 3000 cm3	DWI	0.783	0.655 to 0.912	0.001	0.672 to 0.882
	Fluid-fluid level	0.676	0.506 to 0.847	0.038	0.588 to 0.794
	Wall thickening	0.676	0.538 to 0.873	0.015	0.588 to 0.824
	Fluid-fluid level or wall thickening	0.853	0.718 to 0.988	<0.001	0.735 to 0.941
	Fluid-fluid level or wall thickening with diameter > 5cm	0.706	0.538 to 0.873	0.015	0.588 to 0.824
Controls with TKLV 3000 to < 5500 cm3	DWI	0.610	0.483 to 0.736	0.092	0.513 to 0.703
	Fluid-fluid level	0.653	0.534 to 0.773	0.019	0.556 to 0.744
	Wall thickening	0.711	0.599 to 0.823	0.001	0.624 to 0.794
	Fluid-fluid level or wall thickening	0.827	0.731 to 0.924	<0.001	0.741 to 0.903
	Fluid-fluid level or wall thickening with diameter > 5cm	0.841	0.751 to 0.931	<0.001	0.762 to 0.913
Controls with TKLV 5500 to <8500 cm3	DWI	0.511	0.362 to 0.660	0.881	0.881 0.426 to 0.594
	Fluid-fluid level	0.674	0.531 to 0.817	0.022	0.563 to 0.794
	Wall thickening	0.614	0.466 to 0.762	0.134	0.506 to 0.729
	Fluid-fluid level or wall thickening	0.737	0.606 to 0.868	0.002	0.626 to 0.846
	Fluid-fluid level or wall thickening with diameter > 5cm	0.811	0.690 to 0.933	<0.001	0.706 to 0.906
Controls with TKLV >=8500 cm3	DWI	0.507	0.355 to 0.660	0.927	0.435 to 0.584
	-fluid level	0.635	0.484 to 0.786	0.083	0.512 to 0.743
	Wall thickening	0.645	0.495 to 0.794	0.063	0.518 to 0.772
	Fluid-fluid level or wall thickening	0.783	0.665 to 0.902	<0.001	0.663 to 0.873
	Fluid-fluid level or wall thickening with diameter > 5cm	0.768	0.639 to 0.897	0.001	0.637 to 0.856

## Discussion

Our MRI diagnostic criteria for cyst infection were highly sensitive because at least one of the four imaging features was seen in every patient with cyst infection. Intracystic gas was specific for cyst infection, so we can make a diagnosis of infected cyst whenever intracystic gas is detected. In addition, when an intracystic fluid-fluid level or wall thickening is seen, the specificity or sensitivity for diagnosis of infected cyst is about 80 % (Table 2). ROC analysis also indicated that a fluid-fluid level or wall thickening was useful for the diagnosis of cyst infection (Table 5). A high intracystic SI on DWI differs from the other three MRI features because its specificity is quite low. A high intracystic SI on DWI could reflect minor bleeding or inflammation because a high intracystic SI on DWI can be seen both in intracystic infection and intracystic hemorrhage [7]. Intracystic bleeding or cyst infection occurred in some of the controls even though it was not documented in their medical records. In addition, minor intracystic bleeding or inflammation might occur in some ADPKD patients. ADPKD patients often complain of abdominal pain that is different from abdominal distension [10–12], and such pain might result from minor intracystic bleeding or inflammation. We also often encounter ADPKD patients with slight elevation of CRP although they do not have obvious cyst infection, and such slight CRP elevation might be due to minor intracystic bleeding or inflammation rather than infection. However, a high SI on DWI is the most frequent feature of an infected cyst and was seen in 88.2 % of the cases, so we should not exclude it from diagnostic criteria for cyst infection. We should not make a diagnosis of infected cyst only from a high intracystic SI on DWI, and abdominal pain at the same location as the cyst plus changes on MRI are needed for diagnosis [7]. Abdominal pain is useful for detecting an infected cyst, but it only occurs in about 60 % of patients with cyst infection and previous MRI data for comparison might be unavailable [7]. The present study also revealed that a cyst diameter >5 cm was useful for detecting severe infection that was likely to need drainage [4, 5]. The specificity of a high SI on DWI was 84.4 % for cysts with a diameter > 5 cm, but its sensitivity was lower at 69.1 %. Therefore, we should assess a combination of abdominal pain, sequential MRI changes, and/or cyst diameter >5 cm with the four MRI features to detect infected cysts that need drainage.

In the present study, specificity for infected cyst decreased as TLKV increased. The reason might be that cyst bleeding or inflammation are likely to occur as TLKV increases. In patients with a TLKV > 8500 cm^3^, the specificity of a fluid-fluid level or wall thickening was only 65.8 %, so these features alone are not useful for diagnosis of cyst infection. In these patients with a TLKV > 8500 cm^3^, the sensitivity of a fluid-fluid level or wall thickening was 72.2 %, for cysts with a diameter > 5 cm, but the specificity was lower at 76.3 % (Tables 3 and 4). Therefore, we should not make a diagnosis of infected cyst only from a fluid-fluid level or wall thickening in patients with massive organomegaly, even if the cyst diameter is > 5 cm. Abdominal pain at the same location as the cyst plus changes on MRI are needed for diagnosis in these patients, too. Previous MRI to compare is unavailable in some patients, however, recurrence of cyst infection is likely occur in patients with huge organomegaly, so previous MRI is usually available in these patients.

**Fig. 4:**
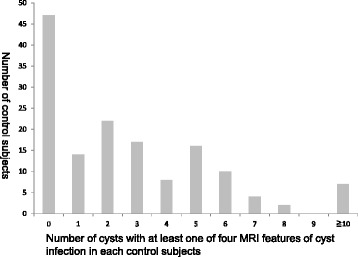
Controls classified by the number of cysts with at least one of the four MRI features of cyst infection

In this study, all controls who had at least one cyst with at least one of the four features of infection among hundreds of cysts were defined as positive, which reduced the specificity of diagnosing infection. However, there were actually not many cysts with at least one feature of infection in each control and only 23 controls (15.6 %) had more than 5 cysts with at least one of the four features of infection (Fig. 4).

An intracystic high SI, fluid-fluid level and sequential changes were only seen on DWI but not on T1WI and T2WI in some cases. One of the reasons for this difference might be that contrast ratio of DWI is higher than that of T1WI or T2WI. Because DWI are useful for evaluating intracystic findings, we suggest DWI should be added to routine MRI in patients with ADPKD. Enhanced CT is associated with exposure to radiation, renal toxicity, and allergy, while enhanced MRI using gadolinium-containing contrast medium is associated with nephrogenic systemic fibrosis, so these imaging methods are not suitable for routine screening. Because it avoids these disadvantages, plain MRI might be suitable as a routine screening method in ADPKD patients with huge kidneys. If we detect abnormal cysts, we should consider diagnoses such as cyst infection, intracystic hemorrhage, or neoplasm. This protocol could potentially improve the outcome of ADPKD patients, but a prospective multicenter study is needed to confirm its usefulness. The optimum interval between abdominal MRI examinations is also controversial, and an appropriate interval should be determined. The present study revealed that intracystic changes on MRI are more likely to occur as the organ volume becomes larger, so we should probably perform MRI more often as organ volume increases.

A limitation of this study is that this was a retrospective study performed at a single center. We enrolled controls who were without cyst infection during the study period according to our medical records, but some of these patients might have been treated for this condition at other hospitals. In addition, selection bias might have occurred because the attending physicians decided whether or not to perform abdominal MRI in the controls. A positive result of cyst contents culture was needed for enrollment of the cases in this study, so our subjects might have had severe cyst infection since bacteria were not eliminated by prior antibiotic therapy. However, it is clinically important to be able to correctly identify the infected cyst in patients with severe cyst infection, because drainage should be performed. The frequency of cysts with a diameter >5 cm was much higher among cases than controls, possibly due to selection bias, since infection may be more likely to become severe if the cyst is large and it could be more technically difficult to drain cysts with a diameter < 5 cm. We determined the cyst/skeletal muscle ratio, but this is less accurate than an apparent diffusion coefficient (ADC) map. However, a longer time is needed to make an ADC map, which is often difficult in patients with ADPKD who can only remain in the supine position for a brief period due to their organomegaly. The MRI protocol that we employed was relatively convenient for the patients, but obtaining 10 mm slices may have reduced the sensitivity of imaging. Finally, the number of patients was not so large, so further investigation is needed.

## Conclusion

A fluid-fluid level or wall thickening on MRI were useful for detecting severely infected cysts in ADPKD patients, showing a sensitivity and specificity of about 80 %. However, the specificity decreased as TLKV increased and was only 65.8 % in patients with massive organomegaly (TLKV > 8500 cm^3^). A cyst diameter >5 cm was useful for detecting infected cysts that needed drainage, and specificity was increased by combining a cyst diameter > 5 cm with the four MRI features of cyst infection. We could identify most cases of severe cyst infection that required drainage by combining the four MRI features with abdominal pain at the same location plus changes from previous MRI findings and a cyst diameter >5 cm. Thus, MRI with DWI is useful for detecting severely infected cysts in ADPKD patients.

## Additional file

Additional file 1: Our diagnostic criteria for cyst infection, acute cyst hemorrhage, and combined cyst hemorrhage and infection. It was published before [7]. (DOC 47 kb)

## Abbreviations

ADC map: Apparent diffusion coefficient map
ADPKD: Autosomal dominant polycystic kidney disease
DWI: Diffusion-weighted images
ESRD: End-stage renal disease
MRI: Magnetic resonance imaging
PET: Positron emission tomography
SI: Signal intensity
TLKV: Total liver and kidney volume
