# Supplementary material for: Intracystic magnetic resonance imaging in patients with autosomal dominant polycystic kidney disease: features of severe cyst infection in a case–control study
Source: BMC Nephrol. 2016 Nov 9;17:170. doi: 10.1186/s12882-016-0381-9 (PMC5103445; doi:10.1186/s12882-016-0381-9)
Supplement: Additional file 1: — Our diagnostic criteria for cyst infection, acute cyst hemorrhage, and combined cyst hemorrhage and infection. It was published before [7]. (DOC 47 kb) [file 12882_2016_381_MOESM1_ESM.doc]

Additional file 1: Intracystic magnetic resonance imaging in patients with autosomal dominant polycystic kidney disease: Features of cyst infection.

Our diagnostic criteria for cyst infection, acute cyst hemorrhage, and combined cyst hemorrhage and infection are presented below.

(Diagnostic criteria for cyst infection)

A-1) No other source of fever detectable

A-2) No evidence for acute cyst hemorrhage (no CT evidence for hemorrhage or no abdominal pain and frank hematuria)

B-1) Maximum body temperature>38°C

B-2) Maximum WBC count>10,000/μl

B-3) Maximum serum CRP>15 mg/dl

C-1) Gas inside the cyst

C-2) High intensity of the cyst on MRI (DWI) (cyst/muscle SI ratio>4 on DWI)

C-3) Fluid-fluid level in the cyst on MRI

C-4) Cyst wall thickening on MRI or CT

D-1) Abdominal pain or tenderness localized to the cyst

D-2) Sequential changes of the cyst on imaging

*For diagnosis of infection, the patient must have both items from A, at least two items from B, at least one item from C, and at least one item from D.

*For a probable diagnosis of infection, the patient requires both items from A, at least one item from B, and at least one item from C.

(Diagnostic criteria for acute cyst hemorrhage)

A) Abdominal pain localized to the cyst and/or frank hematuria

B-1) Maximum body temperature<38°C

B-2) Maximum WBC count<10,000/μl

B-3) Maximum serum CRP<15 mg/dl

C-1) Irregular high-density mass (hematoma) inside the cyst with a density>25 HU on CT

C-2) Cyst density>25 HU on CT

*For diagnosis of hemorrhage, the patient must have A, all three items from B, and at least one item from C.

(Diagnostic criteria for combined cyst hemorrhage and infection)

A-1) No other source of fever detectable

A-2) Abdominal pain localized to the cyst and/or frank hematuria

B-1) Maximum body temperature>38°C

B-2) Maximum WBC count>10,000/μl

B-3) Maximum serum CRP>15 mg/dl

C) Irregular high-density mass (hematoma) with a density>25 HU inside the cyst on CT and/or cyst density>25 HU

*For diagnosis of combined hemorrhage/infection, the patient must have both items from A, at least two items from B, and C.

*For a probable diagnosis, the patient requires both items from A, at least one item from B, and C.
